# Climatic Niche Dynamics of the Astereae Lineage and *Haplopappus* Species Distribution following Amphitropical Long-Distance Dispersal

**DOI:** 10.3390/plants12142721

**Published:** 2023-07-21

**Authors:** Marcelo R. Rosas, Ricardo A. Segovia, Pablo C. Guerrero

**Affiliations:** 1 Departamento de Botánica, Facultad de Ciencias Naturales & Oceanográficas, Universidad de Concepción, Casilla 160C, Concepcion 4030000, Chile; 2 Institute of Ecology and Biodiversity (IEB-Chile.cl), Victoria 631, Barrio Universitario, Concepcion 4030000, Chile; 3 Millennium Institute Biodiversity of Antarctic and Sub-Antarctic Ecosystems (BASE), Santiago 7800003, Chile

**Keywords:** amphitropical disjunction, Asteraceae, biogeographic patterns, Chile, climates, Mediterranean biome, niche conservatism

## Abstract

The tribe Astereae (Asteraceae) displays an American Amphitropical Disjunction. To understand the eco-evolutionary dynamics associated with a long-distance dispersal event and subsequent colonization of extratropical South America, we compared the climatic and geographic distributions of South American species with their closest North American relatives, focusing on the diverse South American Astereae genus, *Haplopappus*. Phylogenetic analysis revealed that two South American genera are closely related to seven North American genera. The climatic niche overlap (D = 0.5) between South and North America exhibits high stability (0.89), low expansion (0.12), and very low unfilling (0.04). The distribution of the North American species predicted the climatic and geographic space occupied by the South American species. In central Chile, *Haplopappus* showed a non-random latitudinal gradient in species richness, with Mediterranean climate variables mainly explaining the variation. Altitudinal patterns indicated peak richness at 600 m, declining at lower and higher elevations. These findings support climatic niche conservatism in shaping *Haplopappus* species distribution and diversity. Two major endemism zones were identified in central Chile and the southern region, with a transitional zone between Mediterranean and Temperate macro-bioclimates. Our results indicate strong niche conservatism following long-distance dispersal and slight niche expansion due to unique climatic variables in each hemisphere.

## 1. Introduction

American amphitropical disjunctions are among the most important biogeographic patterns in plants [1,2]. South and North American amphitropical areas share an important proportion of families, genera, and species connecting floras of different biogeographic origins at shallow and deep time [3,4]. For example, the flora of Chile (southern extratropics) contains 156 out of 716 genera with amphitropical disjunctions [5]. While vicariant disjunctions would have resulted from ancient range disruptions during the Mesozoic, Cretaceous, or Paleogene periods, disjunctions caused by (non-anthropogenic) long-distance dispersal would have occurred during the Late Pliocene or Pleistocene epochs [4]. Analogous climates in South and North American extratropical areas may be an important factor in the success of lineage dispersal and, hence, in the amphitropical biogeographic pattern [1]. However, there are few studies that have directly assessed ecoevolutionary processes within the amphitropical lineages of the Americas.

Niche conservatism is a broadly documented phenomenon [6,7] that refers to the retention of niche-related ecological traits over evolutionary time [8,9,10,11]. Although many plant lineages tend to maintain unchanged environmental conditions in which they can persist and reproduce [9], a long period since colonization may result in the adaptations necessary to inhabit even some of the harshest environments [12]. Climatic niche conservatism impacts species diversification, especially when it is associated with geographical separation [10]. Moreover, niche conservatism has also been shown to play an essential role in long-distance dispersal success, which is critical for the expansion of species ranges and the formation of new biotic communities [9,13,14]. Niche conservatism increases the likelihood of a species successfully colonizing new areas with similar ecological conditions, known as climatic analogues, due to a prior adaptation to the specific environmental conditions. Therefore, they can quickly establish and compete with resident species. This can result in the displacement of native species and the formation of novel ecological communities [13]. However, certain lineages tend to undergo specific climatic niche transitions [15] and, amphitropical disjunctions may be key to understanding the likelihood of the climatic niche shifts, as they may suffer from the founder effect and may be exposed to different environmental conditions after long-distance dispersal [16,17]. In American amphitropical disjunctions, climatic niche shift has been described at a species level in 25 species of natural distribution [18] and niche conservatism has been only detected in invasive species [19].

Within the large and cosmopolitan family Asteraceae, the tribe Astereae, with an estimated 222 genera, is monophyletic and resolves into a number of large clades, including a basal African grade, with disjunct Chinese, South American, and New Zealand lineages, and a polytomy of crown groups in Australasia, South America, and North America [20]. This latter group, referred to as the “North American lineage”, is monophyletic, and most of its genera are endemic or near-endemic to the North American hemisphere. The clades correspond approximately to the subtribes described by morphology [21], and include the subtribe Machaerantherinae, a well-defined, monophyletic group of about 17 genera of the North American taxa. *Haplopappus* Cass., a genus exclusively distributed in South America, is nested within this subtribe, as well as *Grindelia* Willd., which has a distribution in both South and North America [20]. *Haplopappus*, with 70 species, is one of the richest genera in the Southern Andes and contributes significantly to the high level of endemism in the central Chile Hotspot [22]. A phylogenetic study of *Grindelia,* the sister genus of *Haplopappus*, which has nearly 25 species in North America and another 25 in South America, estimated the age of the common ancestor with *Haplopappus* to be about 2.06 Ma (1.12–3.47 Ma) [23], possibly diverging after a single event of long-distance dispersal followed by diversification and geographic expansion in South America [24]

The North American origin of South American clades (*Grindelia* and *Haplopappus*) and the subsequent recent diversification after long-distance dispersal raise questions about the niche dynamics throughout lineage evolution. In this paper, we investigate the eco-evolutionary dynamic of the long-distance dispersal event in the Astereae tribe by comparing the climatic niches of lineages from the original and colonized regions. Additionally, we evaluate the distribution patterns of species richness and endemism in the colonizing lineage *Haplopappus* to shed light on the ecological processes that generated and maintain its high diversity. Therefore, our study aims to address two questions: (i) Have South American Astereae species conserved their climatic niche compared to their North American closest relatives? and (ii) What is the species richness structure of *Haplopappus*, the most diverse genus within the South American Astereae?

## 2. Results

### 2.1. Identification of Sister Lineages

We concatenated ETS and ITS1&2 markers and aligned a sequence matrix for 124 species. The total length of the matrix was 1184 bp, of which 367 were variable for the full matrix and 337 for the ingroup (Table 1). Of the aligned concatenated matrix, the plastid non-coding marker ETS contributed 539 bp (38% of variable sites), while the plastid non-coding ITS1&2 contributed 645 bp (35% of variable sites).

**Figure 1:**
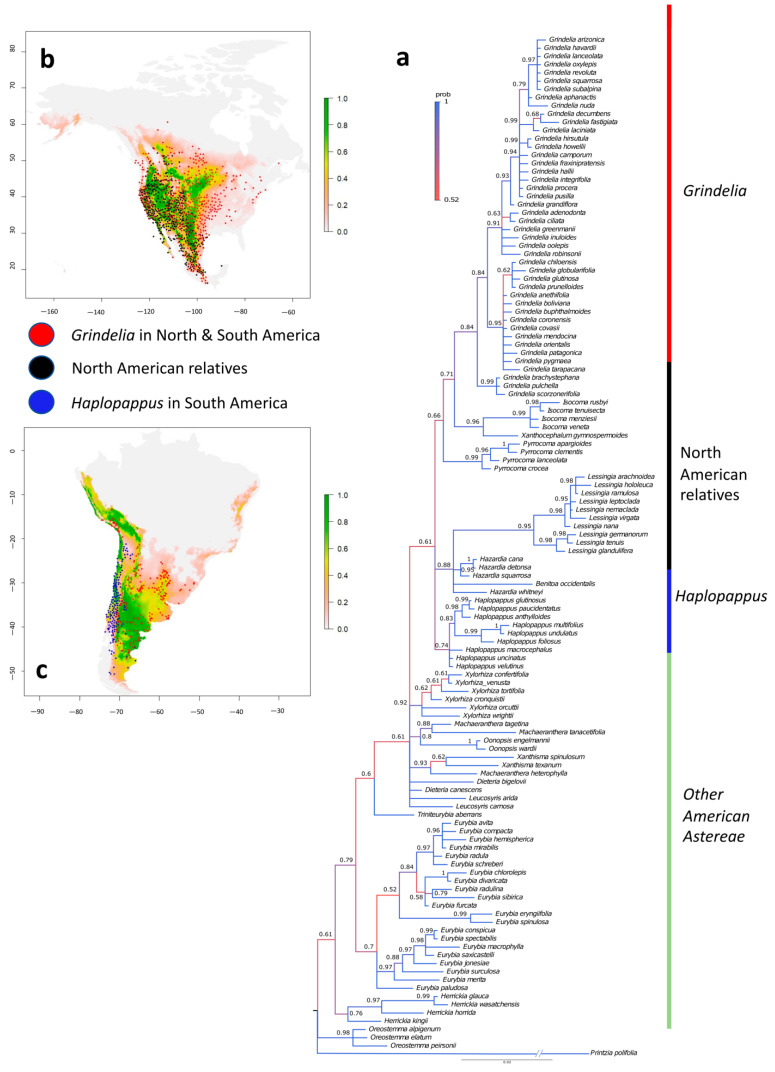
(**a**) Molecular phylogenetic cladogram of Tribe Astereae genera distributed in America based on two loci (plastid markers) for 124 species. Bayesian posterior probabilities are shown at the nodes and reflected in the color of the branches (from dark blue = 1 to red = 0.5). The vertical line describes monophyletic lineages, i.e., red (*Grindelia*), black (North American genera), blue (*Haplopappus*) and green (other American Astereae). Predicted geographic distributions of the disjunct amphitropical clade in the Americas. Colored dots represent occurrences of *Grindelia* species in North and South America (red), North American relatives (black) and South American specie (blue). (**b**) North American niche and occurrences. (**c**) South American niche and occurrences.

Bayesian phylogenetic inference yielded a well-supported phylogenetic hypothesis for the “North American clade” in the Astereae tribe (Figure 1a). The topology of the phylogenetic reconstruction supports the monophyly of the clade, which includes the South American genera *Haplopappus* and *Grindelia*. This monophyletic clade reveals the sister lineages for *Haplopappus* and branches out into three clades in a polytomy (Figure 1a). One of the branches, representing the genus *Haplopappus*, has a good ML value of support (0.74). The other two branches, which have a higher likelihood of being sisters to *Haplopappus*, include seven genera scattered across the branches with varying ML values of support. The second of these branches, with a good ML value of support (0.88), includes groups that are only inhabitants in North America, namely the genera *Hazardia* Greene (13 species), *Lessingia* Cham. (9 species), and *Benitoa* D.D.Keck (one species). The third branch contains the North American genera *Isocoma* Nutt. (16 species), *Pyrrocoma* Hook. (14 species), *Xanthocephalum* Willd. (six species), and representatives of *Grindelia* from both North and South America.

### 2.2. Climatic Niche Comparisons

**Figure 2:**
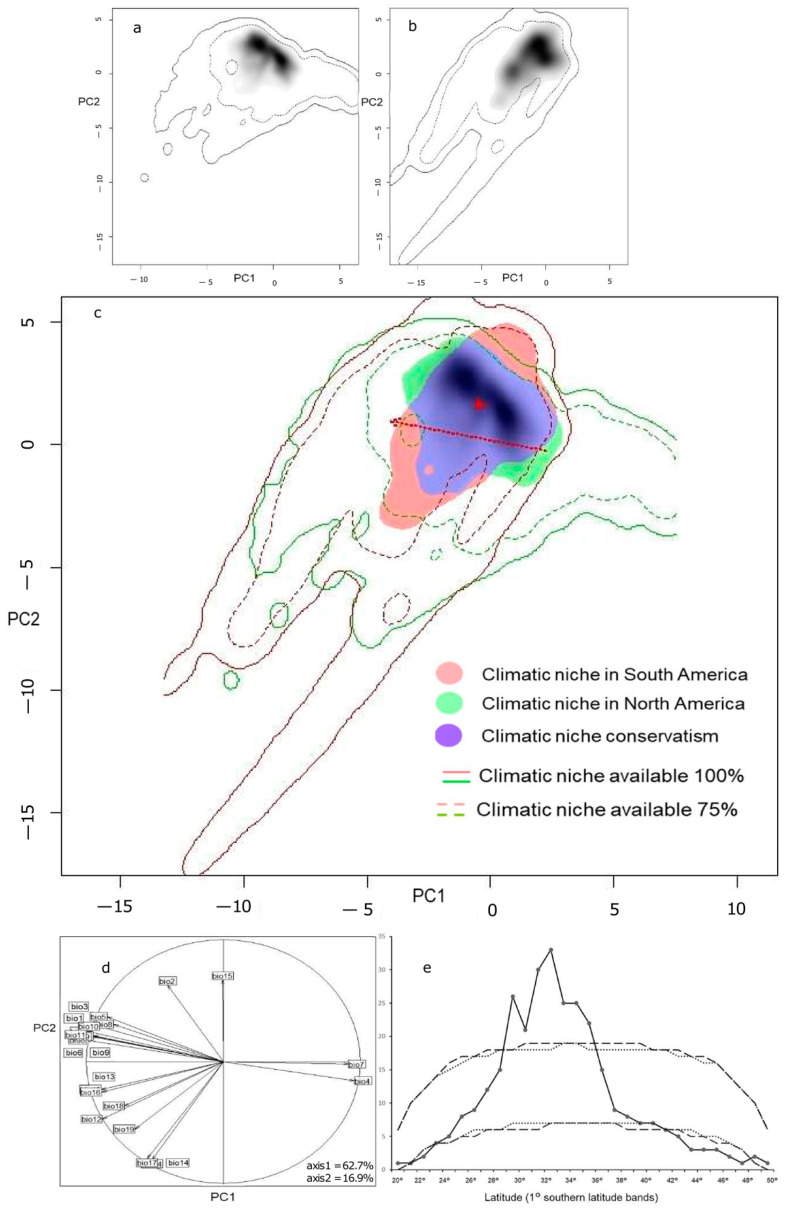
Principal Component Analysis (PCA). PC1 accounts for 62.7% of the data variance, while PC2 explains 16.9%. The climatic niches of North American and South American lineages are depicted in panels (**a**,**b**), respectively, with gray shading representing the density of occurrences. In panel (**c**), a biplot displays the climatic niche of *Haplopappus* and *Grindelia* in South America (red), their sister lineages in North America (green), and blue color indicates overlapping niches (niche conservatism). The dotted red arrow illustrates the shift of the climatic niche centroid from North America to South America, while the solid red arrow represents the displacement of the North American distribution centroid towards the South American distribution. (**d**) Correlation circle for the contribution of variables to the analysis, hidden labels are provided with an additional label. (**e**) Species richness found in each band of 1° latitude (black line with data points). The black dashed lines show the 95% prediction curves of sampling without replacement, and the dotted grey lines with replacement.

We conducted an occurrence search, which resulted in 10,758 records. After applying a 1 km proximity threshold, we retained 3353 occurrences (Figure 1). A Principal Component Analysis revealed two main axes, explaining 79.66% of the total variance *(*Figure 1b,c). An analysis of the climate niche dynamics showed a remarkable overlap between the South American and North American niches (Figure 2c). The corrected Schoener overlap index (D = 0.495) and the uncorrected index (D = 0.737) were significantly higher than expected by chance. The South American niche exhibited moderate expansion in two areas. The centroid of climatic conditions in North America shifted towards the new conditions in South America along the PC1 axis, while the centroids of the lineage distributions in North America shifted slightly but in the direction of the PC2 axis within the invaded area of South America (Figure 2c).

The niche dynamics index indicated a stability index of 0.863, an expanded niche index of 0.136, and an unfilling niche index of 0.044. The similarity and the equivalence tests supported the idea that niches within each region were more similar to each other than expected by chance (*p* < 0.001), compared to randomly simulated niches in the other region. However, niche expansion in South America occurred in two areas. One area expanded towards a group of bioclimatic variables associated with the seasonality of precipitation, while the second area expanded in the opposite direction, correlated with the seasonality of precipitation and the annual temperature range. Notably, there was an important portion of the climatic niche in North America that remained unoccupied by South American lineages, particularly in climates with wider diurnal and annual temperature variations (Figure 2c).

### 2.3. Richness Structure and Explanatory Variables

**Figure 3:**
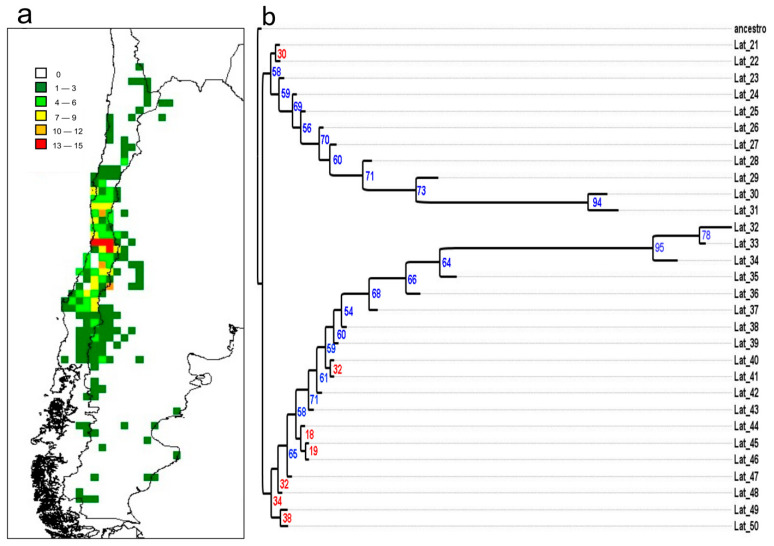
(**a**) Spatial distribution of species richness in *Haplopappus* in South America in 0.5 × 0.5 degree latitude/longitude grid cells. Occurrence data were compiled from Klingenberg [22] and our own field observations. (**b**) Bayesian Analysis of Endemism of the distribution of *Haplopappus* species in 1-degree latitudinal bands. Bayesian posterior probabilities are shown before branches (blue > 50%, red < 50%). Latitude values are on the right and are common to both figures.

The monophyletic *Haplopappus* lineage in South America is geographically distributed in a narrow latitudinal belt, with the center of its species richness located in central Chile, between 30° and 34° S (Figure 3a). Latitudinal and altitudinal patterns in *Haplopappus* species richness showed a structured pattern. The latitudinal pattern in species richness displayed a partial mid-domain effect (Figure 2e). Comparisons of the empirical data with 95% prediction curves of simulations, both with and without replacement, showed that 53% (16 out of 30 points) of the empirical richness records fell within the predicted range of the stochastic analytical null model. Deviations from the simulation analysis occurred in the central part of the distribution range, between 30° S and 37° S, a region of high richness and endemism, where the species accumulation exceeded the 95% distribution prediction limits of the geometric constraint model. Additionally, the species distribution mildly deviated from the model’s prediction in the southern distribution (44° S–49° S) in Argentinian Patagonia and in the north of Chile (23° S), presenting less richness at both ends. A nesting analysis showed that the species richness latitudinal distribution in *Haplopappus* was significantly nested, with the NODF index value being 20.28, higher than the expected value of 9.88 with 95% confidence interval between 9.07 and 10.75, and *p* < 0.0001.

Bayesian analysis of endemism, in which branch lengths are longer in the regions of higher endemism, highlights two distinct and well-supported large areas of endemism (Figure 3b). These areas are separated in the center of richness, where, at the same time, the greatest number of endemic species is accumulated. The northern area ranges between 21° S and 31° S, and the southern zone between 32° S and 50° S. In the phylogram, longer branch lengths are found in the center of the range, and branch length decreases sharply towards the extreme north and south. The southern area shows the highest levels of endemism between 32° S and 34° S, while the northern area shows the highest endemism levels between 30° S and 31° S.

The species richness data showed the highest value per cell with 15 species at 33° S (Figure 2e and Figure 3a), and a gradual decline in richness northward and southward. The Simultaneous Autoregressive (SAR) model explains 47% of the variance (F = 3.072, *p* < 0.005) for the seven independent explanatory variables, two of which were significant in the regression analysis: seasonality of temperature and mean temperature of the wettest quarter (Table 2). Altitude did not have a significant effect on species richness variation per cell.

## 3. Discussion

Our results confirmed the monophyly of the clade containing the genera *Haplopappus* and *Grindelia* in South America and 6 other genera from North America (Figure 1a). We also revealed that the climatic niches of the North and South American species largely overlap and are stable (Figure 2a), implying niche conservatism on a large scale. We found that *Haplopappus* and *Grindelia* in South America occupy a climatic space similar to that occupied by their North American sister lineage despite long-distance dispersal. However, we also detected niche expansion in two opposite directions (Figure 2b,c). Temperature seasonality and the mean temperature of the wettest quarter, consistent with the Mediterranean climate [25,26], were the primary climatic variables explaining the variation in species richness in the diverse South American genus *Haplopappus* (Table 2).

Bayesian phylogenetic inferences yielded a consensus molecular-based tree with branches with different node support. The topology of the phylogenetic reconstruction identified the North America genera *Hazardia, Lessingia, Benitoa, Isocoma, Pyrrocoma, Xanthocephalum,* and *Grindelia* as sister lineages of the South American genera *Haplopappus* and *Grindelia*. The phylogenetic tree is structured into three main subclades. The first is the genus Haplopappus, which is restricted to South America, although the data are limited to only 9 of the 70 species. The second, exclusive to North America, group three genera *Lessingia, Hazardia,* and *Benitoa.* The third, mostly North American, contains genera *Pyrrocoma, Xanthocephalum*, and *Isocoma*, with *Grindelia* grouping the North and South American species in different subclades. *Grindelia*’s distribution in both continents suggests an independent long-distance amphitropical dispersal. Although a previous phylogeny constructed for *Grindelia* [23] has similarities to the topology presented here, it also has differences. While these authors placed *Pyrrocoma* in a sister clade to *Grindelia*, the phylogenetic reconstruction presented in this study suggests that *Pyrrocoma* is nested as a basal lineage in the branch containing *Grindelia* species. The phylogeny of *Grindelia* [23] separates the North American species from the South American species similarly to our analysis. Therefore, both phylogenetic analyses are highly consistent with each other, except for the position of *Pyrrocoma*, an exclusively North American genus.

Our findings provide strong evidence for significant climatic niche conservatism between the South American species in the *Haplopappus* and *Grindelia* genera and their North American sister lineages, with a moderate expansion to unique climatic variables in South America that are not found in North America (Figure 2a,c). The climatic niche analyses revealed a significant overlap between the two regions, and almost no change in the centroid of the lineage distribution (Figure 2c), indicating that *Haplopappus* and *Grindelia* have colonized a range of habitats and climates, as predicted by the projected climate niche of their close North American relatives. This suggests that climatic niche conservatism has constrained geographic spread within analogous climates between both subcontinents, showing a high level of distribution equilibrium [27]. A similar pattern was previously found in the invasive herb *Eschscholzia californica* Cham. (Papaveraceae) which would have reached equilibrium in a hundred-years span [19].

The occupation of habitats in South America is likely favored by climates analogous to those of the northern subcontinent and the more stable climate of the southern subcontinent, where oceanic influences mitigate the thermal and precipitation extremes found in North America [28]. The displacement of the climate background space centroid of the North American lineages compared to the South American lineages’ centroid, oriented along the PC1 axis (Figure 2c), supports the existence of different climates. While the North American climate has a wide range of annual temperature variation, from very cold winters to hot summers, the South American climate is more temperate, probably due to the buffering effect of the ocean [24]. These results suggest that the North American clades occupy a niche space characterized by greater seasonality and a broader range of temperatures than those of the southern clades.

The expansion of the climatic niche in South America is moderate and occurs in two different climatic dimensions (Figure 2c). On the one hand, the climatic niche of the South American group of species is expanding towards conditions characterized by coordinated precipitation and temperature seasonality, along with a lower mean diurnal range of temperatures. On the other hand, the climatic niche is also expanding in the opposite direction, towards climates characterized by a higher seasonality of precipitation and a higher mean diurnal range of temperatures. The former conditions are found in the Mediterranean climate of Central Chile [25,28], while the latter are typical of the Patagonian steppe and the high-altitude regions of the southern Andes, which are characterized by a monsoon climate [29].

The biogeographic pattern of the American Amphitropical Disjunction has long been of interest to botanists [1,2,30]. A collection of 130 examples has distinguished three classes of amphitropical disjunctions [1]: a bipolar pattern with 29 examples, a pattern for temperate lineages with 95 cases, and numerous cases of desert disjunctions [1]. Some amphitropical disjunctions occur between the same or closely related species, as is the case with *Tiquilia* Pers. [31] and *Osmorhiza* Raf. [32]. Other disjunctions occur in well-differentiated groups at the genus level on both continents, such as *Astragalus* L. [33], *Ephedra* L. [34], *Hoffmannseggia* Cav. [35], *Larrea* Cav. [36], among others (see a detailed review at [37]).

The explanatory mechanisms for American Amphitropical Disjunctions have been elucidated for *Tiquilia* using phylogenetic reconstructions for the three major lineages, with at least four long-distance dispersal events required to explain their current distribution [31]. A phylogenetic analysis of the genus *Hoffmannseggia*, which comprises 21 species with an amphitropic distribution in North and South America, revealed a South American origin for the lineage, followed by an initial lineage division and independent dispersals from South to North America for each clade. These dispersals occurred at different times, implying that the pattern is not the result of a single, simultaneous set of dispersals [35]. *Lupinus* L. in America is a well-documented case, with approximately 260 species demonstrating an unusually high altitudinal and latitudinal range as well as a rapid diversification rate in plants [38]. Phylogenetic data indicate a well-supported monophyly of the large Andean *Lupinus* clade, and its position as a sister group to the North American species suggests that its diversification occurred after a single colonization event from North America. The authors propose that the rapid diversification of *Lupinus* on the high Andean plateau was not due to any obvious morphological or physiological traits in the genus, but rather due to the availability of ecological opportunities created by the emergence of large habitats after the orogenic uplift and Pleistocene glaciations. In the species-rich genus *Astragalus*, a phylogenetic study supports two clades of the South American species nested within the North American species. This indicates two separate invasions from North to South America. The South American clades have very recent mean ages but are still significantly different (1.89 and 0.98 My). The rates of diversification are very high for both clades, thus confirming the occurrence of a rapid radiation of plants in the South American *Astragalus*. This finding contributes to the growing list of recent rapid radiations of plants in areas with high physiographic diversity, such as the Andes [33].

Eco-evolutionary dynamics associated with long-distance dispersal have been studied in several regions, with evidence for both niche conservatism and niche shifts within lineages. A niche dynamics analysis for biogeographic disjunctions was performed on 283 Eastern Asian and 91 Eastern North American disjunct plants [14] where niche conservatism was reported. The authors found a significant negative relationship between niche overlap and divergence times in pairwise species disjunctions, indicating niche conservatism following long-distance dispersal and subsequent climatic niche expansion among these species. Likewise, a study was conducted to evaluate potential niche shifts between the North and South American regions in five bipolar species within the *Carex* L. genus. The study used niche overlap analyses to determine the degree of climatic niche expansion and occupancy between the two regions. Results indicated high climatic niche expansion and low levels of niche occupancy between North and South America, suggesting frequent climatic niche shifts during the colonization of South America. Most species have shifted their niche from those occupied in the North to colonize new environments in the South. The observed niche shifts appear to be consistent with the time since colonization [39]. In a study conducted on 25 species of desert plants with an American amphitropical distribution, climatic niche shifts were observed in 24 out of 25 species studied. Most of these species exhibited a displacement towards cooler and more productive environments in South America [18].

Analyses of *Haplopappus* richness patterns provide compelling evidence for a non-random latitudinal gradient in species richness, partially influenced by geometric constraints. Specifically, the highest levels of species richness and endemism occurs in the mid-latitudes of the entire range, spanning from 30° to 35° S. This species concentration is primarily found in Mediterranean Chile, with species richness decreasing in harsh habitats for shrubs, such as the hyperarid Atacama Desert and southern temperate forests. The altitudinal pattern of species richness shows a higher accumulation of species at 600 m, followed by a monotonic decrease in richness towards both lower and higher elevations. This suggests that Mediterranean forests, which have a higher tree cover than mid-elevation habitats, along with alpine habitats, may pose environmental constraints to the geographic spread of this South American group of species. These latitudinal and elevational mid-patterns are consistent with the idea of climatic niche conservatism structuring these patterns, as species are more likely to persist in environments to which they are already adapted. Therefore, the concentration of species richness in specific geographic regions with similar climatic conditions supports the idea that climatic niche conservatism may be playing a role in shaping the distribution and diversity of the *Haplopappus* species. This pattern of high diversity in the Mediterranean zones of Chile among xerophytic species has also been observed in cacti and monocotyledonous geophytes [40,41].

Furthermore, we detected a pronounced nesting pattern in *Haplopappus* species richness, which is indicative of a source-sink dynamic [42]. This dynamic might be primarily driven by the interplay between extinction processes and subsequent recolonization, moving from favorable and species-rich habitats to adjacent environments that are less optimal and have fewer species. [43]. Large-scale climate oscillations associated with Pleistocene glacial-interglacial periods may have induced extinctions in the Atacama Desert due to major vegetation changes [44].

The study of endemism reveals the presence of two major zones of endemism, one situated to the north and the other to the south of the area with maximum endemism and richness (Figure 3b). The region of highest endemism is situated in Central Chile, spanning from 30° to 35° South latitude, in a Mediterranean macro bioclimate, with marked seasonality, characterized by winter precipitation (with the highest precipitation occurring during the coldest quarter of the year) and summer drought (with the lowest precipitation during the warmest quarter). This region experiences a period of aridity of a minimum of two consecutive months [25]. To the north of this region of high endemism, a warm tropical macro-bioclimate develops, characterized by the extensive coastal desert of Peru-Chile. This macro-bioclimate stretches up to 300 km between the coast and the Andes and ranges from 5°S to 30° S. In the southern region of the area of maximum richness, the Mediterranean macro-bioclimate undergoes a gradual transformation into the Temperate macro-bioclimate, marked by the absence of a summer period of water deficit lasting for at least two consecutive months. This transition zone, located at the interface of both macro-bioclimates, corresponds to the Sub-Mediterranean variant of the Temperate macro-bioclimate, which is characterized by a mild summer water deficit of two consecutive months [25]. This Temperate macro-bioclimate demarcates the southern boundary of the distribution of *Haplopappus* along the western margin of the Los Andes Mountain range. In the eastern region of the Andes there is a dry region, where the eastern slope of the Andes is experiencing forced subsidence, resulting in notably dry conditions in Argentine Patagonia [29]. This arid zone contributes to the distribution of a limited number of *Haplopappus* species. Our analysis suggests that the expansion of two distinct lineages into new habitats, originating from a more climatically suitable dispersal center, is associated with the seasonality of temperature and the mean temperature during the wettest quarter, both of which are characteristics of the Mediterranean biome.

Gaining insight into the similarities and differences in environmental spaces among species through time is crucial in comprehending diversity patterns [7] and identifying the mechanisms or processes that drive them [7,13]. The results presented here contribute to the understanding of the eco-evolutionary processes behind the American pattern of amphitropical species distribution, allowing us to improve our understanding of the origin and maintenance of biodiversity in southern South America.

## 4. Materials and Methods

### 4.1. Identification of Happlopapus Sister Lineages

To gain insight into the closely related sister lineages of *Haplopappus*, a search was conducted for Astereae genera found in America using the comprehensive treatment of the tribe [21] and the global phylogenetic revision of the tribe [20]. For the identified genera, a manual search was conducted for available sequences in GeneBank [45], resulting in the identification of five genes for a 499 species, including ETS, ITS1&2, rbcl, trnk_matK, and trnl_lf. The downloaded sequences were assembled and edited using Geneious Prime^®^ 2020.2.3 (Biomatters Ltd., Auckland, New Zealand), followed by manual verification. To assess saturation, the Xia model was applied in DAMBE [46], while Tajima test in software DnaSP version 6.12 [47] was used to test for neutrality. The nucleotide evolution model that best fit our data was determined using TREEFINDER (version March 2011), which resulted in GTR + G. Exploratory phylogenetic analyses were conducted on the matrix using the program raxmlGUI 2.0 v.2.0.6 [48]. The search for an optimal ML tree was conducted with a rapid bootstrap analysis involving 100 trees and 1000 replicates. To provide an outgroup, *Printzia polifolia*, a basal lineage of the tribe Astereae, was utilized. This analysis revealed that the ETS and ITS1and2 genes were the most informative and had the strongest support. We focused on the ‘North American Lineage’ clade, in which Haplopappus and its closely related species are nested, according to the global phylogeny of the tribe [20]. We created a concatenated matrix of the two most informative markers for 124 species including this clade, and each marker was aligned separately and then concatenated (Table 1). The outgroup used for phylogenetic analyses was the species Printzia polifolia. We used PartitionFinder v.2.1.1 [49] with the “-raxml” command line option [50] to search for the best partitioning strategy and the best molecular evolution models for the molecular data set. These analyses used the potential partitions that were defined a priori. The GTR model was selected by PartitionFinder as the best model for the ETS and ITS1&2 markers.

A Bayesian inference analysis was carried out on this matrix using MrBayes v.3.2.7 [51], and unlinked rate heterogeneity, based on the frequencies and substitution rates across partitions. Bayesian ran 30 million generations across four independent runs with four chains each, sampling every 1000 generations. The best models were GTRG for both markers. Convergence was monitored using the standard deviation of the split frequencies, and when this value stabilized below 0.01, it was considered a strong indication of convergence. The associated likelihood values, effective sample size (ESS) values, and burn-in values of the different runs were verified with the program Tracer v.1.7.1 [52]. A consensus tree and posterior supporting data of the nodes were visualized, rooted, and edited using software FigTree v.1.4.4 [53].

### 4.2. Climate Niche Dynamics

To investigate the eco-evolutionary dynamics of the long-distance dispersal event, we compare the climatic niches of lineages from the original and colonized regions. Our chosen approach is to focus on the climatic niche because of its predictive capabilities, as demonstrated by species distribution models. Although geomorphology and soil variables are also important for species distribution, we have excluded them from this climatic niche analysis due to the uncertainty surrounding the geographical location of the majority of the sites under consideration. Although the resolution of climate data, obtained at 1 × 1 km, was considered satisfactory, it did not meet the requirements for soil data, which are available at a more refined resolution of 100 to 300 m, as exemplified by resources such as soilgrids.org.

The georeferenced occurrences of the sister genera *Benitoa* [54], *Grindelia* [55], *Hazardia* [56], *Isocoma* [57], *Lessingia* [58], *Pyrrocoma* [59], *Xanthocephalum* [60] in North America, as well as the occurrences of the Grindelia species in South America, were obtained from the Global Biodiversity Information Facility (GBIF) using the ‘rgbif’ v. 3.7.2 package [61]. Access to the GBIF platform was obtained on 04-21-2022. The downloaded data files underwent a rigorous filtering process; only entries classified as “preserved specimens” from valid herbariums listed under “Institution Code” were retained. The dataset was cleaned for geographic anomalous entries [62] and for environmental outliers [63]. The data are provided in a table (Table S1 in Supplementary Materials) with information on genus, species, latitude, longitude, year of collection, base of record, and herbarium. Climatic data were the standard (19) BioClim bioclimatic variables in WorldClim version 2 [64] for a resolution of 30 s.

Climatic niche characterization and niche comparisons between the North and South American groups were based on Guisan [17] and were carried out in the R language [65] with the Ecospat 3.5 package [66]. Ecospat functions were used to compare niches between the North and South American sister lineages [67,68]. For analyses, the occurrences closer than 1 km were removed. Parameters used were R (grid resolution) = 600, th.sp (threshold species) = 0.05 to remove 5% of marginal occurrences, and equivalence and similarity test repetitions rep = 500. The lineage niches were compared in multidimensional space. Niche overlap was calculated using Schoener’s *D* index [69], which calculates the overlap values of species from the two continents (0 = no overlap, 1 = complete overlap). Based on *D* index, niche equivalence and niche similarity between hemispheres were performed [70]. The equivalence test determines whether compared niches are different by comparing the observed D value against the D values obtained from a distribution of D values (n = 1000; α = 0.05), which are calculated from two random samples taken from the set of all occurrences. The niche similarity test determines whether two niches are more similar than expected by chance by comparing the niche in one region with randomly simulated niches in the other region. Niche overlap can be disentangled in stability, expansion, and unfilling categories. Stability was estimated as the proportion of occurrences of each hemisphere that was in the niche overlap space in the multivariate space. Expansion was estimated by occurrences that are outside the overlap zone, and thus has a unique niche in each hemisphere. Unfilling was estimated by the projected niche in the other hemisphere not yet occupied by the species. This decomposition provides more information about the drivers of niche dynamics between two sister lineages that have evolved different niches [17,71]

### 4.3. Richness Structure and Explanatory Variables

For this analysis, the *Haplopappus* occurrence data were obtained from multiple sources. Firstly, a total of 939 specimens referenced in L. Klingenberg’s 2007 monograph [22] were utilized. In addition, 180 specimens were collected by M. Rosas since 2004. Finally, 12 specimens were included from material deposited in the CONC and ULS herbaria in Chile. The compiled occurrences are presented in Table S2 in the Supplementary Material. This table provides comprehensive details, including species identification, locality, collector, collector number, date of collection, herbarium, and geographical information such as latitude, longitude, and elevation. Occurrence data were refined to a geographic uncertainty of less than 1 km using satellite images, travel diaries [72], travel notes [73], and collectors’ field notebooks [74]. To determine the geographical area travelled through during the period of specimen collection (whether on foot or on horseback), we examined the herbarium notebooks in detail, checking the localities corresponding to the preceding and subsequent numbered entries, or we reconstructed the historical route and explored the contemporary travel routes of the time. Latitudinal distribution patterns were evaluated from a matrix of *Haplopappus* species in latitudinal bands of 1° latitude and absent or present taxa. Continuous distribution was assumed in case of missing intermediate data among latitudes. Species richness (alpha diversity) was calculated as the sum of all species within a latitudinal band. Altitudinal patterns were determined in a similar way using a presence/absence matrix, with rows corresponding to 200-m altitude bands and columns corresponding to species. The species richness in altitudinal band corresponds to the sum of the row and the species altitudinal distribution range corresponds to the sum of columns.

Hypothesis of mid-domain geometric constraints also was evaluated by comparing observed patterns of species richness with simulated curves constructed by a null model using the Mid-Domain Null program with Monte Carlo simulation [75]. Simulated curves were based on real size ranges within a limited domain using stochastic models [76]. We used 50,000 Monte Carlo simulation samples of observed range sizes without replacement (randomization procedure) and with replacement (bootstrap method) to calculate the simulated prediction curves with a 95% confidence. Also, to test if the structure of the species richness patterns is not due to chance, a SIMPROF (Similarity Profile) test was performed using a permutation procedure in software PRIMER 6 v.6.1.2 [77].

The source-sink hypothesis also was evaluated [42] in the nesting analysis that assesses the degree of order in a species set, through a simple species-site presence-absence matrix, when comparing them against an appropriate null hypothesis [78]. A nested distribution will result in non-random distribution patterns, where rare species will only be present at the most species-rich sites, while the most common species will be present at all sites [43]. To estimate the degree of nesting, we used NODF’s index [79]. The statistical significance for the nesting index NODF was evaluated generating a null model from the Monte Carlo algorithm, comparing observed values against a random probability distribution. The null model used in our case was of fixed rows and equiprobable columns, where totals observed by rows are maintained, but totals of columns vary randomly [80]. To generate a frequency distribution of the null data, 50,000 iterations were performed. A nesting analysis was performed with the NODF program [81] and for ordered matrix we used the NED program [82].

Additionally, we analyzed endemism patterns using a Bayesian analysis of endemism (BAE) with the Monte Carlo Markov Chain method used to classify areas based on their shared endemic taxa. Species data by band produces a phylogram that represents nested area sets, where the terminal branches correspond to a latitudinal band of endemism. Trees were rooted to hypothetical areas coded exclusively with absences. Likelihood values and area relationship hypotheses were generated using the M2P model with non-reversible time (i.e., directional) using the BayesPhylogeny 1.0 software [83]. This model uses non-reversible presence-absence data where the rate of change from 0 to 1 is different from the rate of change from 1 to 0 and corresponds to the number of species gained in areas (branches of tree), that result from the ecological processes of immigration or the evolutionary processes of speciation. The process used one million trees sampled every 1000 trees to ensure independence in successive samples. A graphical detection of the convergence zone of the Markov chain was carried out at Maximum Likelihood values and all trees prior to the convergence zone were eliminated. With the approximately 1000 trees selected with Maximum Likelihood values, a consensus tree was built using the BayesTrees 1.3 software [84] and edited with the FigTree 1.4.4 [53].

To study the underlying relationships between species richness and climatic variables, we used a species richness grid with a cell of 0.5° × 0.5° latitude/longitude and corresponds to the species number present in each cell, which approximates the density of the species [85]. In this analysis, we seek to reduce spatial autocorrelation to detect the influence of climate and altitude to determine variation in species richness. Climatic variables were those 19 Bioclim from WordClim2 database [64], all were evaluated by autocorrelation to select independent ones. Simultaneous autoregressive models (SAR) were run to estimate regression coefficients and simultaneously evaluate spatial autocorrelation of each climatic variable together with the interaction of annual mean temperature and annual precipitation [86]. The geographic analyzes were carried out with the DIVA-GIS 7.5.0 software [87] and the regression analyzes with the SAM 4.0 software [86].

## Figures and Tables

**Table 1:** Statistics for the 124 sample DNA sequence alignments for the two loci used (ETS and ITS1&) and for the concatenated matrix.

Locus	Total Length	Ingroup Variable Characters	Total Variable Characters	Parsimony Informative Characters	Ingroup Coverage (%)	Outgroup Coverage (%)	% Variability
ETS	539	128	128	63	1	98	35
ITS1&2	645	94	209	123	1	99	57
Concat. Matrix	1184	337	367	197			

**Table 2:** Parameter estimates for seven independent explanatory variables in the simultaneous autoregressive model (SAR) accounting for 47% of the variance. Seasonality of temperature and mean temperature of the wettest quarter were significant in the regression analysis with *p*-value of less than 0.05%.

Variables	SAR Coeff.
Mean Diurnal Range (Bio2)	0.465
Isothermality (Bio3)	0.156
Temperature Seasonality (Bio4)	0.036 *
Temperature Annual Range (Bio7)	0.852
Annual Mean Temperature (Bio1)	0.192
Mean Temperature of Wettest Quarter (Bio8)	0.181 *
Annual Precipitation (Bio12)	0.001
R^2^	0.469

* *p* ≤ 0.05.

## Data Availability

The datasets presented in this study can be found in online repositories. The names of the repository/repositories and accession number(s) can be found at the following link: https://github.com/M-Rosas/Rosas_etal_Plants_2023 (accessed on 15 May 2023).

## Supplementary Materials

The following supporting information can be downloaded at: https://www.mdpi.com/article/10.3390/plants12142721/s1, Table S1: Species occurrences obtained from GBIF, accepting only records labelled as “preserved specimen”; Table S2: *Haplopappus* occurrences.
